# The Planning of Cottage Hospitals

**Published:** 1921-04-02

**Authors:** 


### The Planning of Cottage Hospitals.

Cottage hospitals formed the subject of a very useful and fully illustrated paper read before the Royal Institute of British Architects on Monday, March 14, by Mr. H. Percy Adams. The classic upon this subject, of course, is Sir Henry Burdett's volume, which ran into several editions, but the last edition appeared as far back as twenty-five years ago. In many rural districts a cottage hospital, or extension of one already in existence, has proved an attractive scheme of war memorial. Promoters of such memorials and the architect to be employed will do well, therefore, to obtain Mr. Adams's excellent paper, in which will be found much suggestive material. The planning of a cottage hospital, however, is a thing apart from the planning of town hospitals, and is beset with perplexities of a difficult nature which are more diffused than in the larger institution. The same unit—the patient's bed—is predominant in both; from this everything radiates, and around it everything in the hospital centres. A cottage hospital cannot be expected to secure the amenities due to and to be found in a general hospital, but it must generally follow in that desirable direction. Committee and staff will be found to be always enthusiastic, but will need to be carefully shepherded by an architect experienced in these all-important matters. Mr. Adams did well to call attention to the dictum so admirably expressed by Dr. Mackintosh in his book on hospital construction, where he says what in effect applies equally to cottage-hospital planning:—

"The main principles underlying the construction of a hospital can no doubt be laid down and applied by any skilful architect. But in a modern hospital there are details of construction of which the needs and the conditions under which the needs can be met are known only as a result of hospital administration. It frequently happens in modern hospital plans that, while the buildings themselves are on approved lines, the lack of administrative detail negatives much of the benefit which might otherwise be obtained."

Much might be said in regard to the selection of the site. In some places we have noticed undesirable sites selected for hospitals. Too much care cannot be devoted to this all-important matter. The site should be freely exposed to the south, with all due consideration for the disposal of placed that it is well away from dusty roadways, and, if possible, on the ideal piece of land, gently sloping towards the south, with all due consideration for the disposal of drainage and sewage. No scheme should be entertained that is likely to cause embarrassment in the matter of future extension. Above all things, the mind must be disabused of dwelling-house ideas, and ornament for its own sake forsworn. It does not follow that a cottage hospital need be unattractive, only that its plan must be the expression of its purpose, and in the hands of a skilful architect this can readily be secured.

